# Cross-linguistically shared and language-specific sound symbolism in novel words elicited by locomotion videos in Japanese and English

**DOI:** 10.1371/journal.pone.0218707

**Published:** 2019-07-10

**Authors:** Noburo Saji, Kimi Akita, Katerina Kantartzis, Sotaro Kita, Mutsumi Imai

**Affiliations:** 1 Kamakura Woman’s University, Kamakura-shi, Kanagawa, Japan; 2 Nagoya University, Nagoya-shi, Aichi, Japan; 3 University of Gloucestershire, Cheltenham, United Kingdom; 4 University of Warwick, Coventry, United Kingdom; 5 Keio University, Fujisawa-shi, Kanagawa, Japan; Hong Kong Polytechnic University, HONG KONG

## Abstract

This paper demonstrates a new quantitative approach to examine cross-linguistically shared and language-specific sound symbolism in languages. Unlike most previous studies taking a hypothesis-testing approach, we employed a data mining approach to uncover unknown sound-symbolic correspondences in the domain of locomotion, without limiting ourselves to pre-determined sound-meaning correspondences. In the experiment, we presented 70 locomotion videos to Japanese and English speakers and asked them to create a sound symbolically matching word for each action. Participants also rated each action on five meaning variables. Multivariate analyses revealed cross-linguistically shared and language-specific sound-meaning correspondences within a single semantic domain. The present research also established that a substantial number of sound-symbolic links emerge from conventionalized form-meaning mappings in the native languages of the speakers.

## Introduction

The arbitrary relationship between sound and meaning has long been considered an important principle of language [1]. However, words whose sounds are motivated by their meanings are widely found across languages. Some languages have a large lexical class of sound-symbolic words called “ideophones,” “expressives,” or “mimetics” [2][3]. Sound symbolism, an iconically motivated link between the sound of a word and its meaning, is not limited to words in this special lexical class. Edward Sapir noted that English speakers associate novel words containing the vowel /i/ with smallness more frequently than words containing /a/ [4]. Another well-known example of sound symbolism is the association between sonorancy and roundness reported by Köhler [5]. When presented with a curvy shape and a spiky shape, most respondents preferred the curvy shape as a referent of *maluma* and the angular shape as a referent of *takete*.

A key unanswered question pertinent to sound symbolism is its universality. There is a host of evidence suggesting that regardless of their native language, people can detect the sound-meaning relationships of the kind Sapir and Köhler reported [6–10]. On the other hand, not every case of sound symbolism was shown to be universally detectable. For example, Iwasaki, Vinson, and Vigliocco examined whether English speakers could detect the meanings of Japanese mimetics (i.e., conventional sound-symbolic words) referring to locomotion, asking English and Japanese speakers to rate the mimetics on a set of semantic-differential scales (e.g., energetic vs. non-energetic; fast vs. slow) [11]. English and Japanese speakers’ ratings agreed on some sound-meaning mappings but not on others: Japanese speakers associated mimetics starting with a voiced consonant (e.g., *dosi-dosi* ‘tramping’) with the meaning component “a big person walking,” while associating those starting with voiceless consonants (e.g., *katu-katu* ‘walking with a clicking sound’) with “feminine” and “formal” styles of walking. Although English speakers mapped voiced consonants to “bigness,” they did not agree on the mapping of voiceless consonants.

More recent studies demonstrated cross-linguistically shared sound symbolism and language-specific sound symbolism are both present within a language. Dingemanse and colleagues examined whether all mimetics are uniformly sound-symbolic across different semantic domains [7]. In their experiment, 208 mimetics were sampled from five semantic domains (sound, motion, texture, shape, and visual appearance) in five languages (Japanese, Korean, Semai, Siwu, and Ewe). Each mimetic was presented to Dutch participants who were not familiar with any of these languages. The participants were then asked to guess the meaning of each word in a forced-choice task. The success rates varied considerably across different semantic domains; the mimetics in the sound domain were easily mapped to the original meanings, but those in other domains (e.g., shape, motion) were harder.

These findings suggest that some semantic domains are more apt for sound symbolism than others. However, other factors such as phonetic features might also affect the accessibility of sound-meaning associations. Shinohara and Kawahara examined how images of size were correlated with three different phonetic factors (voicing of obstruents, vowel backness, and vowel height) in four languages (Chinese, English, Japanese, and Korean) [12]. They reported that vowel backness was associated with largeness in all languages; in contrast, voicing contributed to the image of largeness in Chinese, English, and Japanese, but not in Korean, suggesting that the accessibility of sound-meaning associations may vary even in widely attested size-sound symbolism.

### Methodological issues to uncover cross-linguistically shared and language-specific sound-meaning correspondences

In this research, we investigate the nature of sound symbolism shared across different languages and sound symbolism specific to a particular language in fine granularity, adopting a multivariate data-mining approach. The majority of previous psychological studies on sound symbolism, including well-established shape-sound symbolism and size-sound symbolism, have been conducted in search for universally shared sound symbolism. In such studies, people’s sensitivity to sound symbolism has been tested mostly using a forced-choice task [13, 14] or a semantic-differential rating task [11, 15–17]. However, these methods are limited in at least three ways when looking for universal and language-specific sound symbolism.

First, it is difficult to a priori determine how many sound patterns and meaning dimensions should be chosen to illuminate the whole system of sound symbolism. The structure of sound symbolism has not been well described for a number of semantic domains. For example, Kawahara and Shinohara argue that abrupt acoustic changes are associated with emotions that involve an abrupt onset (e.g., shock, surprise) in the same way that such sounds are readily connected to abrupt changes of the directions of lines (i.e., shape-sound symbolism) [18].However, these studies examined only a limited number of sound-meaning links.

Second, it is not clear at what level of abstraction sound and meaning should be analyzed [19]. A majority of studies on sound symbolism have adopted phonetic features as a unit of sound in their sound-symbolic analysis [12, 20, 21], but some researchers have used larger or smaller units of sound, such as the mora (e.g., /ma/) and the phonestheme (e.g., *gl-* in vision/light-related words in English, such as *glance*, *glimmer*, *glisten*, and *glitter*) [13, 22, 23]. A similar problem can be noted for the analysis of meaning as well. For example, most studies on shape-sound symbolism have discussed the “spikiness” or “curviness” of shapes [5]. However, Kantartzis [24] and Kawahara & Shinohara [12] argue that sounds are mapped to high-order semantic features like “abruptness,” a concept that covers abrupt changes across a diverse range of semantic dimensions such as shape, color, and emotional state. This means that, when we examine the correspondences between sounds and meanings, it is not clear on what basis particular semantic dimensions should be singled out.

Third, when participants were asked to choose a sound-symbolically matching word for a given visual stimulus in a forced-choice task, their success rates were greatly affected by particular sounds used in the target word and the foil [7, 20]. For example, Ramachandran and Hubbard reported that 95% of their participants mapped *bouba* onto a rounded shape, and *kiki* to an angular shape [14]. However, when different word pairs were used (e.g., *tage* for an angular shape and *yame* for a rounded shape), the agreement rate sharply dropped to 70% [18].

To circumvent these limitations and uncover latent sound symbolism, we propose a new methodology in which a bottom-up approach which explores what kinds of sounds and meanings are linked in a language and a top-down hypothesis-testing approach, which tested whether the links detected by the bottom-up exploration are shared (or not shared) across different languages. One possible way of a bottom-up search for sound symbolism would be a large-scale corpus/dictionaries investigation. Blasi, Wichmann, Hammarström, Stadler and Christiansen, for example, apply this approach to a list of 100 words from 6452 languages [25]. They found that a considerable proportion of basic words tended to bear specific sound segments. Furthermore, the sound-meaning associations uncovered in the study included the associations that had not been reported in previous research (e.g., the association between *r* sounds and round shape).

In the present study, we propose a different bottom-up approach, which used a production-elicitation task, where participants created words that best describe a given set of visual stimuli. One advantage of the production-elicitation method over the corpus-based method is that it allows us to investigate the relationship between sounds and meanings in a target semantic domain directly and in much finer ways than the corpus-based approach. Since participants can use any possible combinations of phonemes, we are able to determine which level of sound properties (e.g., the mora or the segment) plays the most critical role in producing sound-symbolic effects without posing any presupposition on the level and unit of sound symbolism both on the sound side and the meaning side. Given a large variation observed in sound-symbolic words across different languages, sounds and meanings are expected to involve many-to-many, rather than one-to-one, mappings [25–27]. As we describe in more detail below, employing the Canonical Correlation Analysis (CCA) enables us to deal with this type of mappings.

### The present study

We aimed to find sound-meaning correspondences native speakers of English and Japanese recruit in the domain of motion. Japanese and English largely differ in the significance of sound-symbolic words in the lexicon. Japanese has a class of mimetic words, which are characterized by a set of morpho-phonological and morpho-syntactic features [2, 28, 29]. They have either monomoraic ((C)V) or bimoraic ((C_1_)V_1_C_2_V_2_) roots, and mostly function as adverbs. Mimetics in Japanese are productive in that novel mimetic words are very often coined to create new sound-symbolic effects. In contrast, English does not have a lexical class dedicated to sound symbolism, although phonesthemes involve non-arbitrary sound-meaning correspondences some scholars regard as sound-symbolic [22, 30, 31, 32]. Moreover, mimetics in English are considered mostly onomatopoeic (e.g., *pop*, *crack*). If we find common sound-meaning mappings between speakers of English and speakers of Japanese despite these differences in their lexical systems, they could be good candidates for broadly applied sound symbolism across different languages in the world.

We chose human locomotion as the domain of our empirical investigation because it is one of the domains in which sound-symbolic words are frequently found across languages, including Basque, Emai, Indonesian, Korean, and Japanese [32–37]. Furthermore, this domain is likely to contain both cross-linguistically shared and language-specific sound symbolism [7]. The participants from both language groups first rated the video clips on five semantic scales (i.e., size, speed, weight, energeticity, and jerkiness); they then generated novel sound-symbolic words for these clips. The analyses were carried out in two steps. In Step 1, by using the Canonical Correlational Analysis, we investigated the systems of sound symbolism in Japanese and English speakers' responses. In Step 2, we tested whether the detected sound-meaning links found in Canonical Correlation Analysis are shared between the two languages by statistical mixed effect models.

## Materials and methods

This study was approved by the ethics committee at Keio University (#24) on July 28, 2009. The written informed consent was obtained from all participants before the experiment.

### Materials

Seventy short video clips of various types of human locomotion (*M* = 7.3 sec, *SD* = 2.7) were created. In each video, a person was walking or running from left to right in a certain manner. Eight Japanese actors, 4 males and 4 females, moved in various manners that were possible exemplars of locomotions that could be expressed by 44 Japanese mimetics (e.g., *burabura* ‘strolling’, *nosinosi* ‘striding heavily’, *tekuteku* ‘walking with light steps’) and 26 English manner-of-motion verbs (e.g., *bustle*, *trot*, *limp*). (As we report in detail later, there was no significant difference in the difficulty for English and Japanese speakers to generate novel words from the videos based on Japanese mimetics and those based on English verbs.)

For the rating task, five 11-point semantic-differential scales (from 1 to 11) were used. The semantic dimensions were “size” (large–small), “speed” (slow–fast), “weight” (heavy–light), “energeticity” (energetic–non-energetic), and “jerkiness” (jerky–smooth). These scales were selected following Iwasaki et al. [11], who also compared sound-symbolic intuitions between English speakers and Japanese speakers in the domain of motion. Iwasaki et al.’s study found that, in Japanese speakers’ semantic evaluation of the Japanese mimetic *tokotoko* ‘trotting quickly with short steps’, steadiness was positively correlated with fastness and energeticity and negatively correlated with the length of strides. It is possible that the semantic dimensions in the current study are correlated with each other in a similar way to Iwasaki et al.’s study and mapped to similar sounds. As we describe later, we will use a multivariate analysis to capture not only whether each of the five meaning variables contributes to motion-sound symbolism, but also how these meaning variables are correlated with one another. This method should allow us to uncover not only sound-meaning mappings but also the relationships among sounds and among meanings.

### Participants and procedure

Thirty Japanese speakers and 27 English speakers, all undergraduate students enrolled in Keio University and the University of Birmingham, respectively, participated in the experiment. The Japanese participants have some knowledge of English, but do not use it regularly and hence were not fluent in it. The English participants did not know Japanese.

The participants in both language groups first saw the 70 videos presented in a random order, and evaluated each of them on the five semantic-differential scales. After the rating task, they watched the videos again in a different randomized order and created a novel sound-symbolic word for each video clip. The participants were required the rating task prior to the word creation task, so that participants would not think that they should rate the semantic dimensions for the meaning of created words rather than the motions. We needed to have each participant do both the rating task and the word creation task because we were interested in seeing the correspondence between each participant's perception of the motion and the sounds s/he used to depict it. The participants were asked to present only one word per video (see Appendix for the precise instruction given in the two languages).

In the current study, both Japanese and English participants were instructed to create CVCV-shaped words that intuitively matched the motion in the video clips. We restricted their responses to the CVCV form, which is familiar to Japanese speakers but less so to English speakers. There were two reasons for this decision. Japanese is far more restricted than English with respect to what consonants are allowed in the coda of a syllable (only /n/ and the first part of a geminate consonant are allowed). Furthermore, Japanese does not allow any consonant cluster in the onset of a syllable. In order to give comparable degrees of freedom to English and Japanese speakers, we limited ourselves to words with two open syllables.

Some readers may be worried that forcing English-speakers to produce words in the unfamiliar CVCV may hinder them from recruiting their natural sense of sound symbolism. However, we found that the phonological pattern of produced words in the current study was virtually the same as that in spoken English in corpora, with the correlation value as high as .83. So we believe that the negative influence from this manipulation was minimum (see Analysis 1 in the Result section below for more details).

The English-speaking participants were additionally asked to pronounce the novel words they typed, as the actual pronunciations of the words might not be obvious from the English-based spellings.

### Data preparation

We obtained 2100 words from Japanese participants and 1890 words from English participants. Both Japanese and English results contained some non-CVCV forms, such as monosyllables (e.g., *ga*, *ten*) and vowel-initial words (e.g., *oho*, *iri*). Also excluded were words that were identical to, or apparently derived from, existing nouns or verbs (e.g., *robo*, created from the noun *robotto* in Japanese or *robot* in English). A total of 1,695 (Japanese) and 1,227 (English) words were retained after the data cleaning procedure and were submitted to analysis.

We analyzed the initial mora (/C_1_V_1_/) of the produced words (e.g., *ka* of *kato*), based on the previous finding that it plays the most important role in sound symbolism (see [18, 27, 38–42] for the demonstration that the first CV plays a primary role in word recognition processes).

The data were coded using Bailey and Hahn’s scheme that captures several phonetic features of consonants and vowels (see Table 1) [43]. Six phonetic features were used for Japanese and English. In Japanese, palatalization (/Cy/) was also considered, as it is phonemically and phonosemantically relevant in Japanese (but not in English). For example, the Japanese mimetic *syurusyuru* ‘moving with a whizzing sound’ is considered as a palatalized counterpart of *surusuru* ‘moving smoothly’; mimetic palatalization is sound-symbolically associated with a diminutive function [28]. The coding was carried out by two native speakers of English and two native speakers of Japanese. All of them majored in psycholinguistics at the graduate school of Keio University or Birmingham University. The results were also checked by two of the second and fourth authors.

**Table 1:** The coding scheme for phonetic features.

	Japanese	English
C_1_ place of articulation	Labial, Velar, Alveolar, Glottal, Palatal	Labial, Velar, Alveolar, Glottal, Palatal
C_1_ sonorancy	Sonorant, Obstruent	Sonorant, Obstruent
C_1_ manner of articulation	Stop, Affricate, Fricative, Glide Nasal, Flap	Stop, Affricate, Fricative, Glide, Nasal, Lateral, Rhotic
C_1_ voicing	Voiced, Voiceless	Voiced, Voiceless
C_1_ palatalization	Palatalized, Not palatalized	-
V_1_ height	High, Mid, Low	High, Mid-high, Mid-low, Low
V_1_ backness	Front, Central, Back	Front, Central, Back

Separate data matrices were prepared for English and Japanese. In each matrix, each row represents a novel word token produced by participants for a given video stimulus, and five columns represent the five meaning variables for the video stimulus. Additional columns represent phonetic features for the word (seven columns for Japanese and six for English). Thus, the data obtained from the Japanese-speaking participants and the English-speaking participants were tallied into a 1695 × 12 matrix and a 1227 × 11 matrix, respectively.

### Validity check

To establish the validity of the data, we first checked whether the number of excluded words was equally distributed over the 70 videos. The average of excluded words per video was 5.8 (*SD* = 3.0) in the Japanese data and 8.5 (*SD* = 1.7) in the English data.

As noted earlier, the number of videos based on English verbs (26) was smaller than the number of videos based on Japanese mimetics (44). This may be a concern if the participants generated novel words by analogy to the words in their native language. If that were the case, they should have produced fewer word types for the videos based on words in their native language because their responses should converge on the variants of the base words. The Japanese speakers produced 22.6 and 21 word types on average for Japanese-based videos and English-based videos, respectively, and the English speakers produced 16.8 and 17.5 word types, respectively. We conducted a mixed-effects Poisson regression model predicting the number of word types produced for the 70 videos, with the participants as a random factor and the base language of the videos (English verbs or Japanese mimetics), participants’ language (English or Japanese), and their interaction as fixed factors. This analysis indicated that Japanese participants produced more word types than English participants (estimate *Beta* value = .19, *z*-value = .19, *p* < .01). However, the effects of neither the base language of the videos nor the interaction involving this factor reached the level of significance (*p*s > .15). Thus, no evidence was found that participants generated words more readily for videos that were based on expressions in their own language.

## Results

### Analysis 1: Descriptive statistics

Before exploring the relation between the sound variables and the meaning variables, we first calculated descriptive statistics for the phonetic features (Table 2). We compared the number of occurrences of each value in each phonetic feature with their distributions in spoken Japanese and English in corpora, using the Corpus of Spoken Japanese [44] for Japanese, and the corpus used in [45] for English. The values in the phonetic features in the present data were distributed in a highly comparable way to those of the Japanese corpus (*r* = .85) and the English corpus (*r* = .83), respectively. This indicates that the participants recruited the inventory of sounds typical of their native languages. It is worth noting that even English speakers who were not familiar to CVCV forms used the sounds that are common in English in creating sound-symbolic words. These results confirm that the participants used sounds in a non-random fashion (specifically, based on the phonological system of their native language) and, therefore, the sound-symbolic words produced in the current study were valid for seeking the speakers’ sound-symbolic intuition.

**Table 2:** The distribution of sounds in the C_1_V_1_ of the produced words in Japanese and English.

Sound	Feature	Value	Frequency in the Japanese data	Frequency in the English data
Consonant	Place of articulation	Alveolar	1037 (61%)	626 (51%)
		Glottal	56 (3%)	64 (5%)
		Labial	287 (17%)	346 (28%)
		Palatal	119 (7%)	95 (8%)
		Velar	196 (12%)	95 (8%)
	Sonorancy	Obstruent	1307 (77%)	818 (67%)
		Sonorant	388 (23%)	408 (33%)
	Manner of articulation	Affricate	95 (6%)	25 (2%)
		Flap	35 (2%)	-
		Fricative	555 (33%)	356 (29%)
		Glide	57 (3%)	113 (9%)
		Lateral	-	90 (7%)
		Nasal	174 (10%)	114 (9%)
		Rhotic	-	91 (7%)
		Stop	779 (46%)	437 (36%)
	Voicing	Voiced	773 (46%)	643 (52%)
		Voiceless	922 (54%)	583 (48%)
	Palatalization	Not palatalized	1437 (85%)	
		Palatalized	258 (15%)	
Vowel	Height	High	546 (32%)	443 (36%)
		Mid-high	-	276 (23%)
		Mid	807 (48%)	-
		Mid-low	-	119 (10%)
		Low	342 (20%)	388 (32%)
	Backness	Back	441 (26%)	654 (53%)
		Central	513 (30%)	-
		Front	741 (44%)	572 (47%)

Note: The numbers in parentheses represent the percentages of observed phonetic values within the phonetic feature categories.

### Analysis 2: Exploratory qualitative approach to uncover covert sound-meaning correspondences in Japanese and English

To investigate the system of sound-meaning correspondences, we conducted a variant of Canonical Correlation Analysis (CCA), which uncovers the structure among categorical variables [46–48]. Like Principle Component Analysis (PCA), CCA reduces the number of dimensions in multivariate space and visualizes implicit structures underlying multiple data sets (e.g., sound and meaning). While PCA can handle only a single set of variables, CCA accommodates two (or more) data sets consisting of different variables, allowing us to examine relationships among variables in two different data sets as well as relations within each data set. In the present study, we used CCA to examine correlations both within and between the sound dataset and the meaning dataset. In other words, we explored not only sound-meaning associations but also correlations within the sound variables and within the meaning variables. If a certain value for a phonetic feature (e.g., alveolar) was correlated with another feature (e.g., fricative), this indicates that the two sounds may form a larger cluster of sound (e.g., alveolar fricatives, such as [s]) to be mapped to a certain meaning feature. The original CCA process assumes that all input variables are measured by a numeric scale, because it adopts Pearson correlations between every observed variable to compute the sum of the eigenvalues of the synthetic variables. However, in the present case, the correlation matrix could not be directly calculated from raw data as the sound variables were categorically coded. So we adopted non-linear CCA proposed by Van der Burg [47, 48]. This method allows us to incorporate nominal variables as the input and convert every categorical variable to a numeric one. This process of quantification is generally called as optimal scaling, in which the optimal quantification for categorical variables and the estimation of synthetic variables are performed simultaneously: the canonical correlation value between the two data-sets are determined comparing the synthetic variables computed from each set with a compromise set of scores assigned to the categorical values in the given data. Consequently, the numeric variables are assigned to each categorical variable, allowing us to interpret the correlation between categorical (i.e., sound variables) and numeric variables (i.e., rating scores). Likewise, some of the meaning variables (e.g., “size” and “weight”) may be correlated with one another to form a larger (more abstract) semantic cluster, such as “magnitude.” In this way, we were able to explore relevant levels of sound and meaning without restricting ourselves to a predetermined set of sound-meaning pairs.

The results of CCA are presented in three steps. First, we present the canonical correlation values which indicate how strongly the sound and meaning variables were tied in the Japanese and English data. Second, we compute the loading scores. These scores enable us to specify what sound and meaning clusters were important in the system of sound-meaning correspondences in both languages. However, the loading scores only show the sound-symbolic correspondences at the level of sound variables, such as “manner of articulation” or “place of articulation”; in other words, they do not tell us which value in a particular variable is associated with which value in a different variable. In the third step, we visualized which meaning clusters are associated with the specific sound value (e.g., “fricative” in manner of articulation; “velar” in place of articulation).

The 1695 (the number of produced words submitted to the analysis) × 12 (7 sound variables and 5 meaning variables) data matrix for the Japanese group and the 1227 × 11 (6 sound variables and 5 meaning variables) matrix for the English group were (separately) fed into the CCA program packaged in IBM SPSS Statistics 20 [49]. We first computed the canonical correlation values. The canonical correlation values are simple Pearson *r*s among synthetic variables, which collapse information from correlated variables to extract a small number of dimensions. In our case, we computed two synthetic values, combining the sound variables (7 for Japanese, 6 for English) and the 5 meaning variables. To estimate how many dimensions should be extracted, we first calculated the canonical correlation values for a four-dimensional solution. The resulting values in Japanese were .49, .29, .25, and .20. Because the decreasing curve is clearly leveling off at Dimension 2, we adopted a 2-dimensional solution with the Japanese data. In contrast, the canonical correlation values in the English data were lower than those in Japanese across the board, and no such clear cut off point was found: the values were .17, .15, .14, and .10. We adopted the two-dimensional solution in the English data so that we could compare the two languages easily. The canonical correlation values of the first and second dimensions were significantly high in both language groups (*r*s = .49 (first dimension) and .29 (second dimension) in Japanese; *r*s = .17 (first dimension) and .15 (second dimension) in English; *p*s < .01). These canonical correlation values indicate the strength of sound-meaning associations. The canonical correlation values of the English group were substantially lower than those of the Japanese group, suggesting that the degree of association between sound and meaning is generally weaker in the English group. This result shows that sound-symbolic intuitions are more stable and consistent in Japanese speakers than in English speakers, presumably because sound-meaning correspondences are much more conventionalized and systematic in Japanese than in English.

To further explore the links between sound and meaning, we next examined the pattern of the component loadings for each of the meaning variables and the sound variables (see Tables 3 and 4 for loading scores in Japanese and English, respectively). Polarity of loadings (positive or negative) of the meaning variables tells us whether a group of meaning variables contributed to a given dimension in the same direction. For example, in the Japanese group, the “size” and “weight” were loaded in the same direction along Dimension 1, which means that the value for size and weight were positively correlated with each other and they were both associated with certain sound variables in the same way, while smallness and lightness would get together in the other direction. As the sound variables were originally categorical, the positivity/negativity of the loading scores in the sound variables were arbitrary and the absolute values counted as the index of the importance of the dimension. The contribution of each sound variable to each dimension broadly indicates how important the given sound variable is for the dimension. For example, “C_1_ manner” and “V_1_ height” obtained a high absolute value (.50 and .60, respectively) in Dimension 2 in Japanese, suggesting that these sound variables were strongly associated with this dimension.

**Table 3:** Component loadings for the Japanese data.

Dataset	Meaning variables	Dimension 1	Dimension 2
Meaning	Size (large–small)	−.50	.38
	Speed (slow–fast)	−.30	−.54
	Weight (heavy–light)	−.81	−.19
	Energeticity (energetic–non-energetic)	.30	.42
	Jerkiness (jerky–smooth)	−.26	.27
Sound	C_1_ place	−.10	−.24
	C_1_ sonorancy	−.32	−.15
	C_1_ manner	.10	.50
	C_1_ voicing	−.80	.05
	C_1_ palatalization	.40	.07
	V_1_ height	−.03	.60
	V_1_ backness	−.13	−.38

Note: The underlined loadings have the absolute value larger than .45, which are considered reliable by Tabachnick and Fidell (2007).

**Table 4:** Component loadings for the English data.

Dataset	Meaning variables	Dimension 1	Dimension 2
Meaning	Size (large–small)	−.40	.12
	Speed (slow–fast)	.56	.06
	Weight (heavy–light)	−.11	.47
	Energeticity (energetic–non-energetic)	−.62	−.32
	Jerkiness (jerky–smooth)	−.09	−.46
Sound	C_1_ place	.04	−.70
	C_1_ sonorancy	.27	.07
	C_1_ manner	.18	−.19
	C_1_ voicing	.58	−.03
	C_1_ palatalization	-	-
	V_1_ height	−.39	−.15
	V_1_ backness	.07	.12

Note: 1) The underlined loadings have the absolute value larger than .45, which are considered reliable by [50]. 2) The dimensions for English have different meanings from the dimensions for Japanese (Table 4).

Based on the criteria proposed by Tabachnick and Fidell, the loading scores higher than .45 are considered to be reliable (the underlined scores in Tables 3 and 5) [50]. As noted above, the pattern of the loading values in the Japanese group shows that the meaning variables “weight” (−.81), “size” (.50), and the phonetic feature “C_1_ voicing” (−.80) received high loadings on the same plane in Dimension 1, suggesting that “weight” and “size” are clustered together to form a semantic unit and are symbolized by the “voicing” feature. Along Dimension 2, the sound features “C_1_ manner” (.50) and “V_1_ height” (.60) and the meaning feature “speed” (.54) were loaded significantly, which yielded the second most important mappings in the observed sound-symbolic system of Japanese.

**Table 5:** Summary of the mixed-effects model for Link (1): “size” and “weight”–“C_1_ voicing”.

Fixed effects	Estimate	Standard error	Df	t-value	p-value
Intercept	−8.73	0.17	92.8	−52.4	***
C_1_ voicing	−.66	0.11	2905.1	−8.8	***
Language	0.11	0.21	50.5	0.54	n.s.
Voicing: Language	−1.91	0.22	2896	−8.61	***

Note:

‘***’ 0.001 ‘**’ 0.01 ‘*’ 0.05 ‘.’ 0.1 ‘ ‘ 1.

In the English group, the semantic features of “speed” (.56), “energeticity” (−.62), and the sound feature of “C_1_ voicing” were heavily loaded on Dimension 1 (see [11]for a similar correlation between “speed” and “energeticity”), while “weight” (.47), “jerkiness” (−.46), and “C_1_ place” (−.70) were loaded heavily on Dimension 2. Like the Japanese group, the contribution of “C_1_ voicing” was the strongest of all. However, the meaning associated with voicing was different between the two languages.

Note that the loading scores only tell us correspondences between the sound features and meaning categories. In other words, they do not specify which sound values were associated with the meanings. To identify specific sound-meaning mappings, we computed the averages of the object scores for each phonetic value and for each dimension (see [46] for details of the algorism). Like the principle component scores in PCA, the object scores in CCA are assigned to each individual CV produced by participants, and represent standardized scores which indicate how each CV included in the produced word is weighted on the extracted dimensions. The averages of object scores across all relevant phonetic values indicate how each phonetic value contributed to the given extracted dimension. For example, for “voiced” and “voiceless” sounds for Dimension 1 in Japanese, we calculated the average of the object scores for Dimension 1 across the 773 voiced-initial words (for “voiced”) and the equivalent average across 922 voiceless-initial words (for “voiceless”) (see Table 2 for the frequencies of each phonetic value).

**Fig 1:**
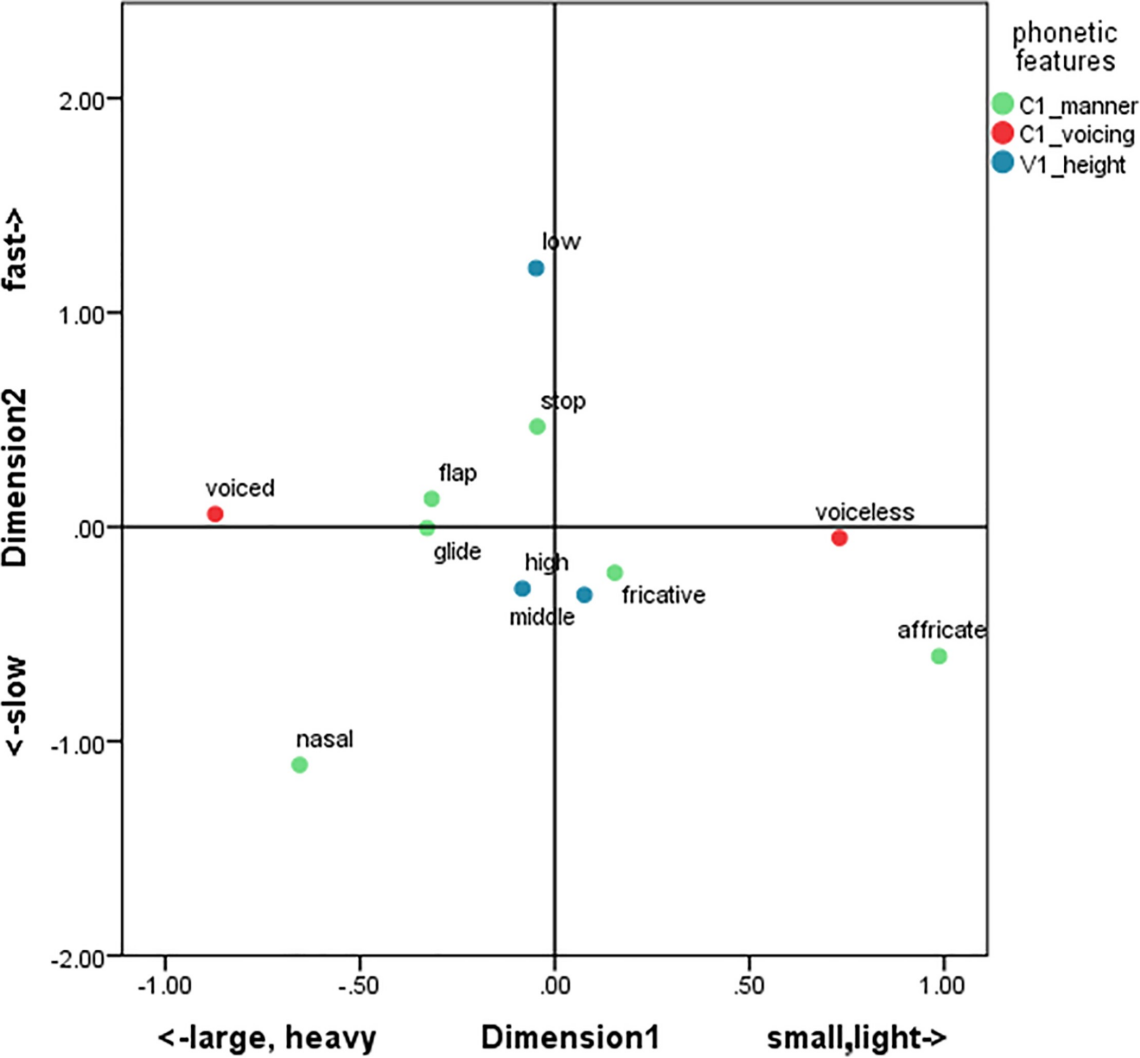
Averages of object scores for individual phonetic values in Japanese.

**Fig 2:**
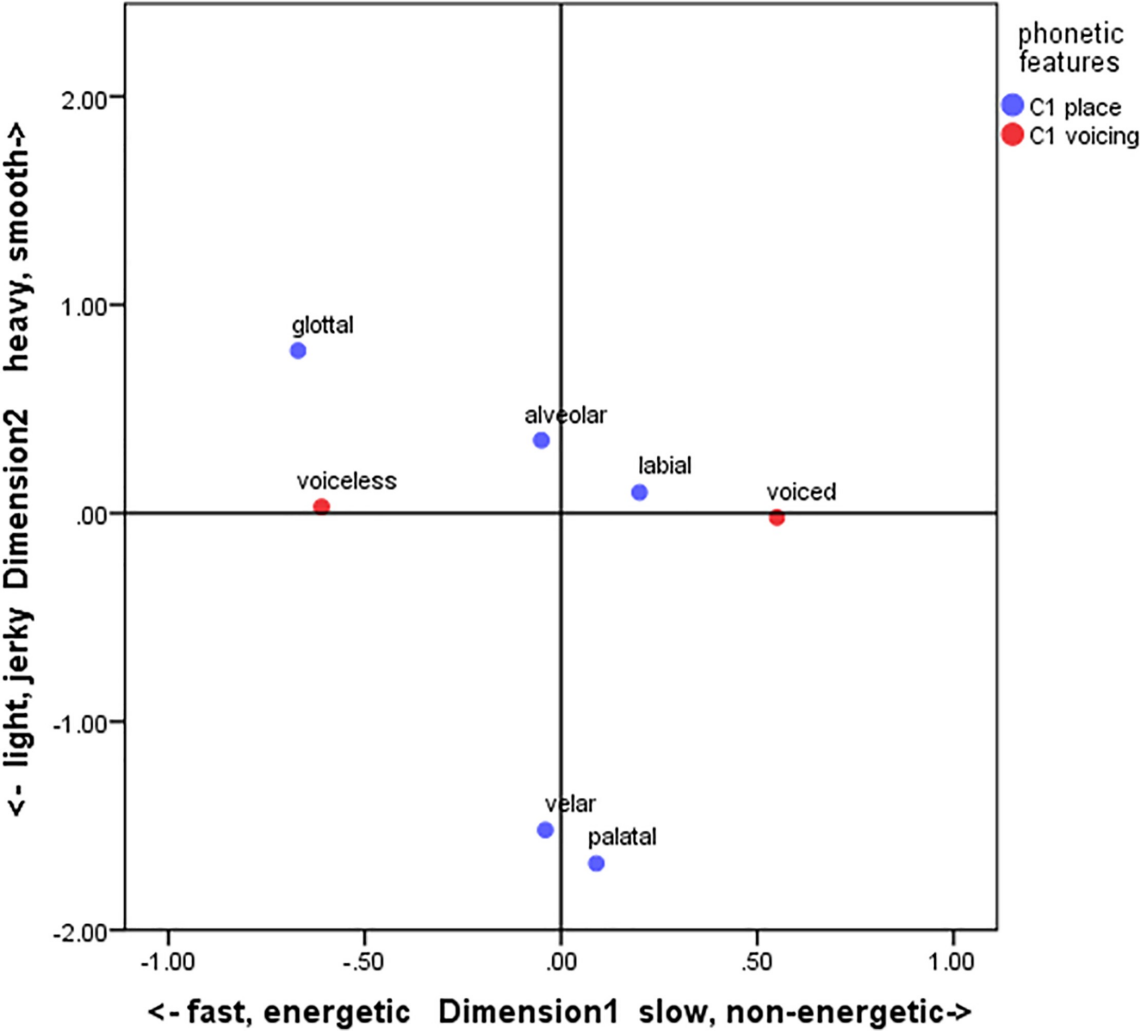
Averages of object scores for individual phonetic values in English.

Each point in Figs 1 and 2 represents the weight of each phonetic value for Dimensions 1 and 2 in Japanese and English, respectively. Note that the points were drawn only for the sound variables for ease of viewing. The relevant meaning variables were shown as the labels for each dimension, since the meaning variables can be interpreted intuitively in light of the polarity of the dimensions (e.g., the negative/positive halves of Dimension 1 linearly correspond to heavy/light meanings).

In Fig 1, the points representing “voiced” and “voiceless” were polarized along this dimension and corresponded to “large”/“heavy” and “small”/“light” motion, respectively. This suggests that voicing is important for the Japanese sound-symbolic system, consistent with the literature [12, 28, 32]. Along Dimension 2, featuring “V_1_ height” and “C_1_ manner,” the “low” vowel (e.g., [a]) was placed on one end (the positive side) and “nasal” consonants (e.g., [n], [m]) on the other, suggesting that the “low” vowel is mapped to *fastness*, and “nasal” to *slowness*.

**Fig 3:**
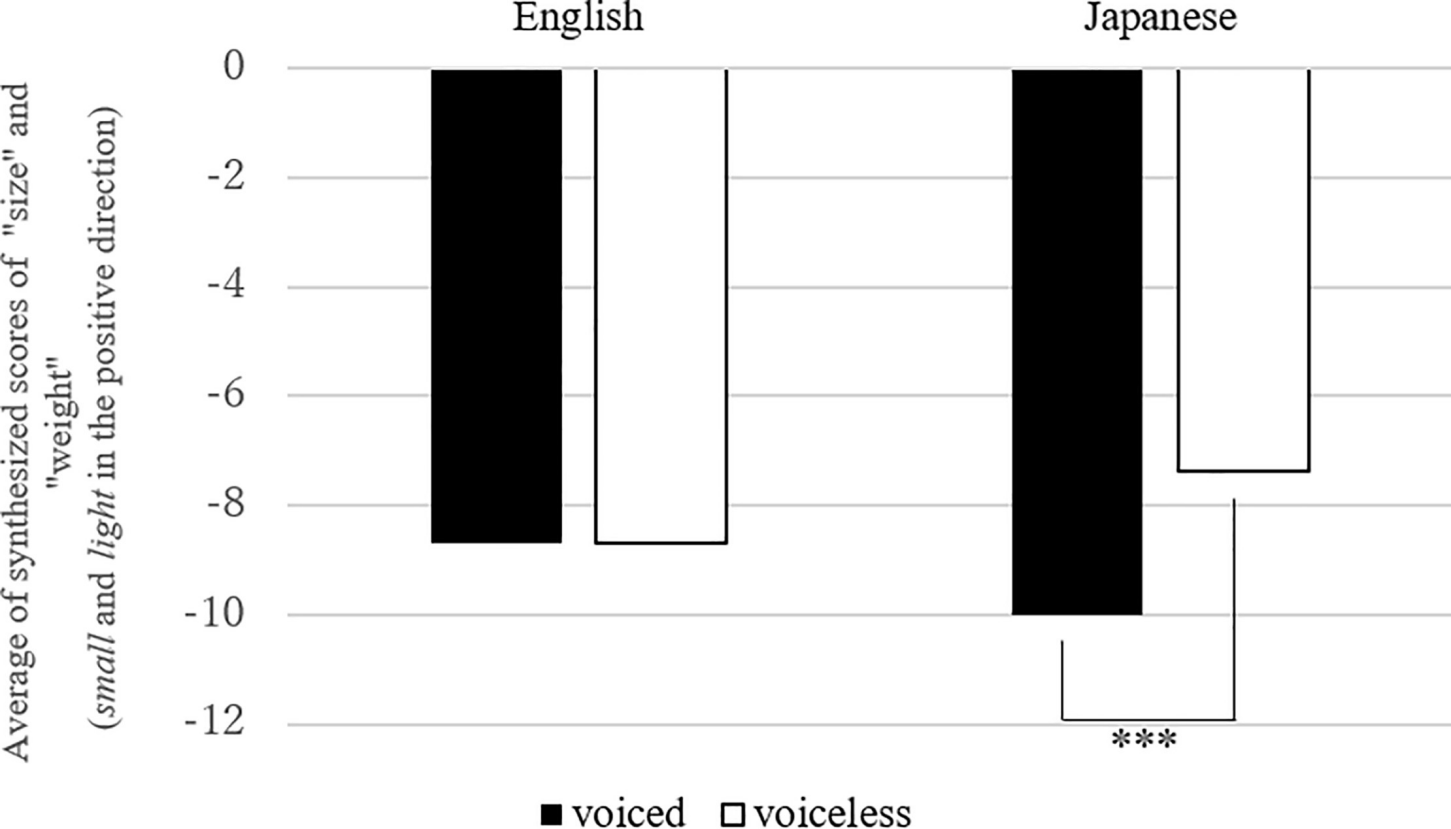
Mean number of synthesized scores of “size” and “weight” in “voiced” and “voiceless” consonants (Link 1). Note: The effect of voicing was significant in Japanese (estimates = 1.62, standard error = .15, df = 2868.2, t.ratio = -10.5, p < .001), indicating that the larger negative scores (i.e., large and heavy motion) were obtained in the condition of the voiced consonants, than in that of the voiceless consonants in the Japanese data.

The results for the English group show both similarities to and differences from the Japanese data (Fig 3). Here, Dimension 1 is characterized by the phonetic feature “C_1_ voicing,” with the “voiced” consonants on the far right (“slow and non-energetic”) and the “voiceless” consonants on the far left (“fast and energetic”). Thus, “C_1_ voicing” plays an important role in symbolizing manner of locomotion in English, similar to the sound-symbolic system in Japanese (see [11], [12], [51] for the similar findings). On Dimension 2, “C_1_ place” showed the heaviest loading (Table 4). Along this dimension, two phonetic features are clearly associated: “palatal,” identified as [j] (e.g., *yupi*), and “velar,” realized as [ɡ] or [k] (e.g., *gaga*, *kachi*), are associated with “light and jerky” motion. Interestingly, the previous studies also reported the sound-symbolic links between “C_1_ place” and jerkiness in English. Barrera-Pardo’s novel-name elicitation task [52], for example, found that velar sounds are often used to depict non-human-like creatures with irregular forms, such as aliens and monsters. Thus, the sound-symbolic links obtained in CCA are at least in part consistent with the previous findings, indicating that the combination of the elicitation/production task and the multivariate analysis is effective and promising in investigations of sound-symbolic systems.

Thus far, CCA uncovered potential sound-symbolic links in Japanese and English separately. The detected sound-meaning links are more strongly connected than the other links within each language. However, these results do not guarantee that the sound-meaning associations observed in one language are equally strong in the other language. In the next step, we statistically test whether the sound-meaning links suggested in one language by CCA is shared by the other language.

### Analysis 3: Commonalities and differences in sound-meaning correspondences between Japanese and English

The following five sound-symbolic links identified in the CCA analysis in either the Japanese or English group were examined to see whether they were shared by the two languages: (Link 1) “size,” “weight,” and “C_1_ voicing”; (Link 2) “speed” and “V_1_ height”; (Link 3) “speed” and “C_1_ manner”; (Link 4) “speed,” “energeticity,” and “C_1_ voicing”; (Link 5) “weight,” “smoothness,” and “C_1_ place.” The former three were found in the Japanese data, while the last two were found in the English data. For each of the five sound-meaning links, we conducted a mixed-effects model with the rating scores for the “meaning” variable (e.g., “speed” for the analyses of Links 2–4) as a dependent variable. The contribution of *sound* (the target phonetic feature), *language* (Japanese/English), and the interaction between the two were examined as fixed effects; the participants and the stimulus videos were included as random effects. We applied centering to the fixed predictors [53]. If a given sound-meaning link is shared between the two languages, the effect of *sound* would not interact with *language*, as the phonetic features alone would explain the variance of the meaning variables. In contrast, if the interaction between *sound* and *language* is found to be significant, the link is likely to be language-specific (at least the link is stronger in one language than the other). When the interaction effect was significant, a post hoc analysis was carried out to determine in which language group the sound significantly contributed to the meaning (lsmean packages for R) [54].

Note that in some cases, multiple semantic scales (e.g., “size” and “weight”) were correlated along a single dimension (Links (1), (4), and (5)). To represent the correlated variables as a single dependent variable, we obtained the synthesized score, which was computed as a linear combination of the loading scores for the relevant semantic features. For example, for Link (1), if the participant rated “size” as “5” and “weight” as “3,” the scores were multiplied by the loading scores of “size” (−.5) and “weight” (−.81) in Japanese (see Table 4), and the resulting synthesized score was obtained as the sum of the weighted values (−4.93).

**Fig 4:**
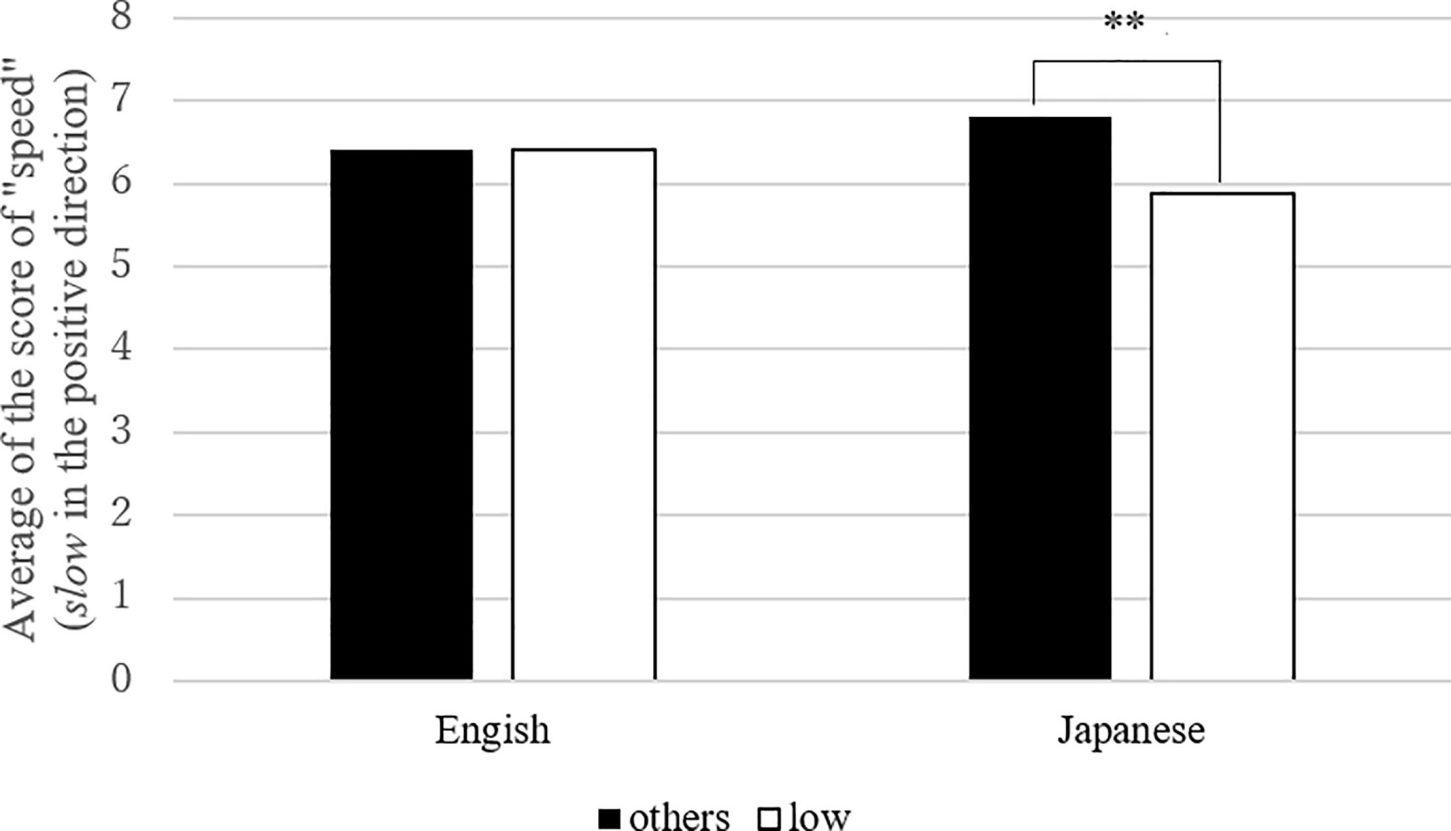
Mean number of “speed” in “low” vowels and the others (Link 2). Note: the effect of low vowel was significant in Japanese (estimates = -0.44, standard error = 0.17, df = 2863.55, t.ratio = -2.6, p < .01).

**Fig 5:**
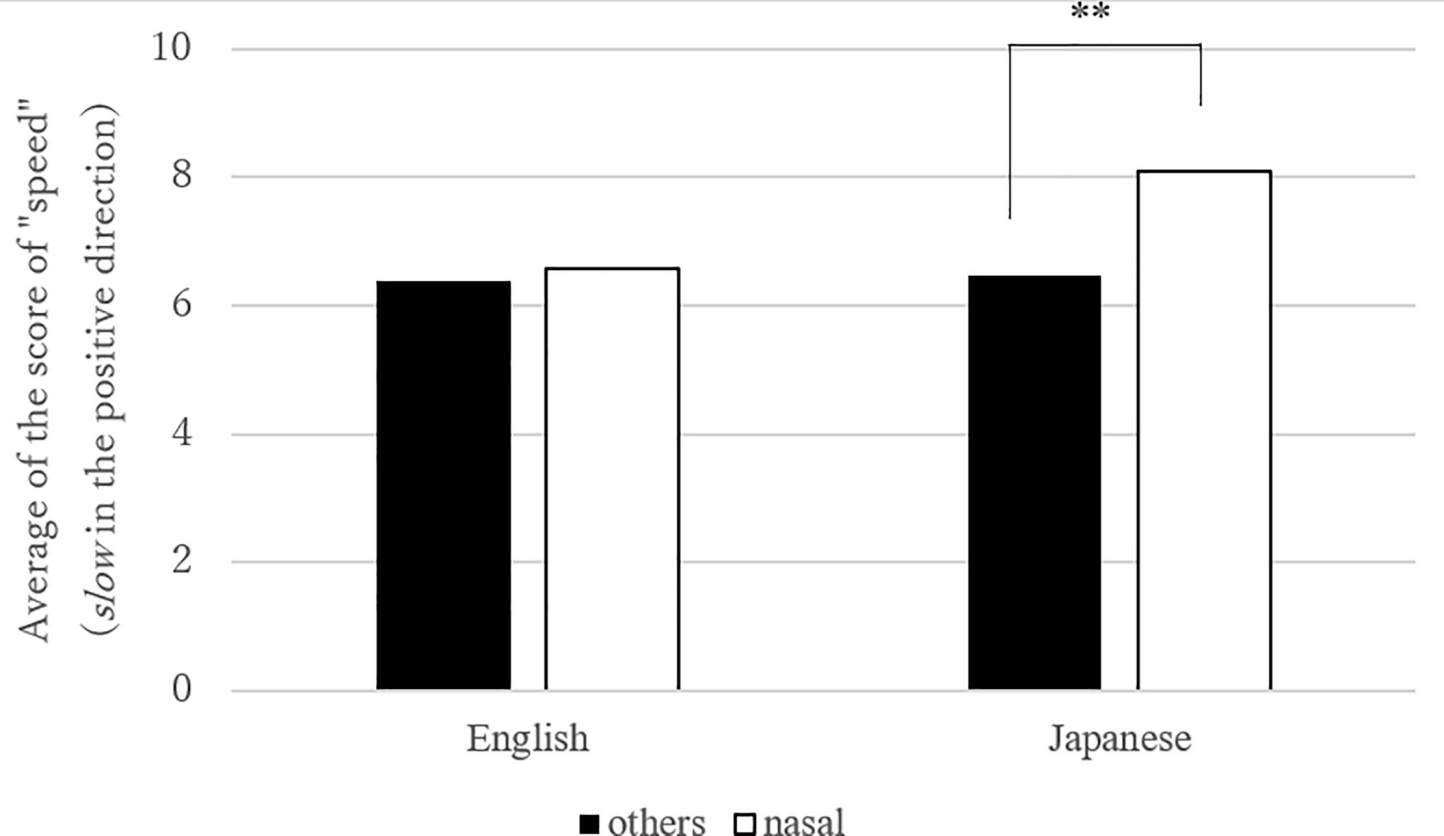
Mean number of “speed” in “nasal” consonants and the others (Link 3). Note: the effect of nasality was significant in Japanese (estimates = 0.68, standard error = 0.22, df = 2876.95, t.ratio = 3.11, p < . 01).

**Fig 6:**
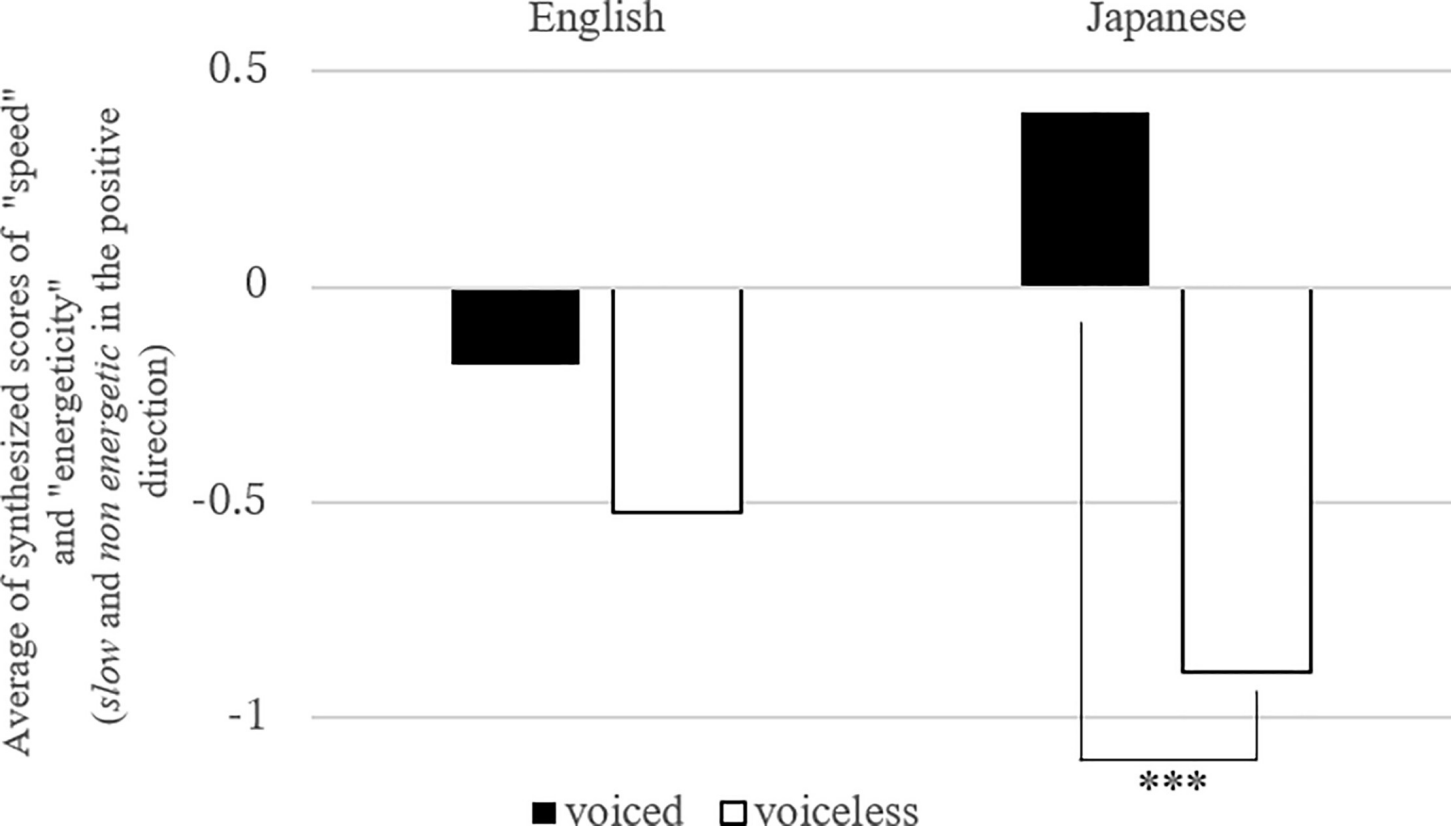
Mean number of synthesized scores of “speed” and “energeticity” in “voiced” and “voiceless” consonants (Link 4). Note: the effect of the voicing was significant in Japanese (estimates = 0.59, standard error = 0.15, df = 2913.59, t.ratio = 4.01, p < .0001).

**Fig 7:**
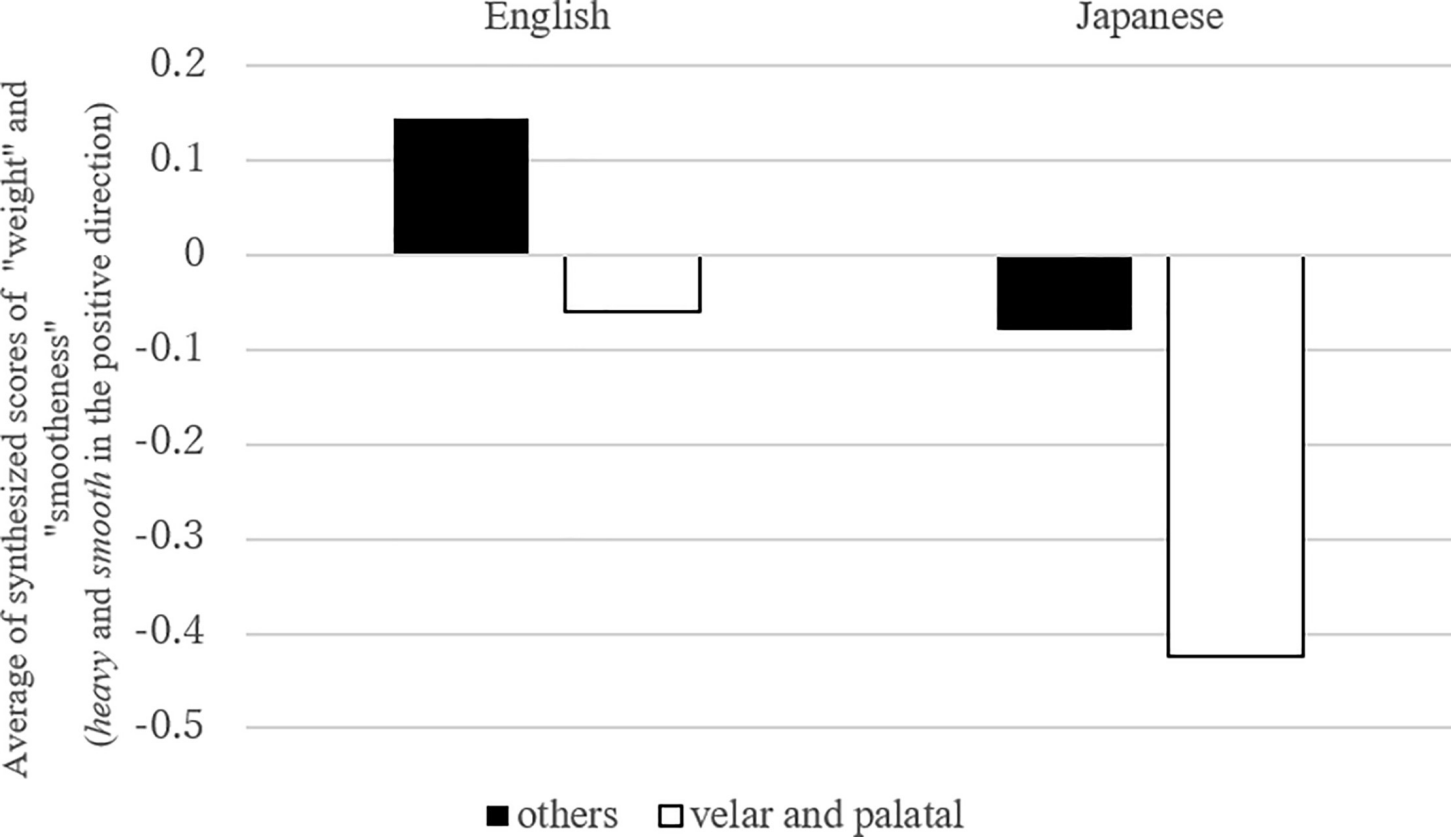
Mean number of synthesized scores of “weight” and “smoothness” in “velar”/”palatal” consonants and the others (Link 5).

Figs 3–7 present the mean synthesized scores for each of the five links (with the results of post-hoc analyses in the figure captions), and Tables 5–9 present the summary of the mixed-effects model for each link. The first model examined Link (1), i.e., the correspondence between “C_1_ voicing” and the synthesis of “size”/“weight” (Fig 3, Table 5). The interaction between *sound* and *language* was significant. A subsequent post-hoc analysis revealed that the effect of the sound (“voiced” and “voiceless” contrast) was significant only in the Japanese group. That is, voiced C_1_ was associated with larger values in the synthesis of “size” and “weight” variables.

**Table 6:** Summary of the mixed-effects model for Link 2): “speed”–“V_1_ height”.

Fixed effects	Estimate	Standard error	Df	t-value	p-value
Intercept	6.48	0.18	85.6	35.7	***
V_1_ height	−0.16	0.12	2873.2	−1.34	n.s.
Language	0.07	0.16	65.8	0.42	n.s.
V_1_ height: Language	−0.56	0.24	2867.7	−2.36	*

‘***’ 0.001 ‘**’ 0.01

‘*’ 0.05 ‘.’ 0.1 ‘ ‘ 1

**Table 7:** Summary of the mixed-effects model for Link 3): “speed”–“C_1_ manner”.

Fixed effects	Estimate	Standard error	Df	t-value	p-value
Intercept	6.62	0.19	107.9	34.595	***
C_1_ manner	0.22	0.17	2876.3	1.3	n.s.
Language	0.59	0.20	161.7	2.88	**
C_1_ manner: Language	0.91	0.34	2864.5	2.67	**

‘***’ 0.001

‘**’ 0.01 ‘*’ 0.05 ‘.’ 0.1 ‘ ‘ 1

**Table 8:** Summary of the mixed-effects model for Link 4): “speed” and “energeticity”–“C_1_ voicing”.

Fixed effects	Estimate	Standard error	Df	t-value	p-value
Intercept	−0.33	0.18	81.9	−1.82	n.s.
C_1_ voicing	0.37	0.11	2909.2	3.33	***
Language	0.05	0.16	50.1	0.32	n.s.
C_1_ voicing: Language	0.46	0.21	2892.8	2.15	*

‘***’ 0.001 ‘**’ 0.01

‘*’ 0.05 ‘.’ 0.1 ‘ ‘ 1

**Table 9:** Summary of the mixed-effects model for Link 5): “weight” and “smoothness”–“C_1_ place”.

Fixed effects	Estimate	Standard error	Df	t-value	p-value
Intercept	−0.09	0.1	115.5	−0.85	n.s.
C_1_ place	−0.22	0.08	2864.2	−2.63	**
Language	−0.25	0.16	67.8	−1.63	n.s.
C_1_ place: Language	−0.13	0.17	2866.9	−0.76	n.s.

‘***’ 0.001

‘**’ 0.01 ‘*’ 0.05 ‘.’ 0.1 ‘ ‘ 1

The next two models (Figs 4 and 5, Tables 6 and 7) examined Link (2) between “speed” and “C_1_ height” (“low” vowel vs. “high/mid” vowels) (Table 6) and Link (3) “speed” and “C_1_ manner” (“nasal” vs. the rest) (Table 7). Again, we found a significant interaction between *language* and *sound* in both cases. Post-hoc analyses revealed that “C_1_ height” and “C_1_ manner” both contributed to the model only in Japanese. That is, low vowels were more readily connected to fast motion than high/mid vowels, and nasal consonants were more strongly associated with slow motion than other consonants in Japanese.

The next analysis (Fig 6, Table 8) examined Link (4), the link between “speed/energeticity” and “C_1_ voicing.” Here again, the significant effect of interaction was revealed. Although the link was first suggested in the English CCA rather than Japanese, the effect of “C_1_ voicing” was not significant in English, but it was in Japanese. Thus, voiced C_1_ was associated with smaller values in the synthesis of “speed” and “energeticity” variables. Finally, the test of Link (5) (Fig 7, Table 9) only revealed the main effect of *sound*, indicating that “velar” or “palatal” consonants were linked to “light” and “jerky” motion in both language groups (Table 9).

## Discussion

This research investigated sound symbolism in the domain of human locomotion in Japanese and English, aiming to uncover sound-meaning mappings that have not been hitherto noted, using a hybrid method which combined an explorative data-mining approach and a hypothesis-testing approach. This methodology offered a new useful way for researchers who wish to explore non-arbitrary relationship between form and meaning in different semantic domains without needing pre-set hypotheses. Because any hypothesis-testing approach requires pre-determined sound-symbolic links (i.e., hypotheses to examine), the majority of studies on sound symbolism have focused on very limited domains, such as shape and size. The method using CCA enables us to overcome this problem and expand the research field, offering a way of hypothesis generation in any semantic domains without limiting ourselves to rely on intuition of our own or of other researchers. However, as it only captures the sound-symbolic pattern within a language, this exploratory approach does not guarantee that the detected links in one language are significant to the same degree in another language. We have overcome this limitation by combining CCA and mixed-effects model analysis for hypothesis testing. In other words, by using the CCA and the mixed model hierarchical analysis, we were able to explore sound-meaning links without pre-set hypotheses but at the same time were able to statistically test whether the identified links would be language-specific or shared across languages.

Cross-linguistically shared and language-specific aspects of sound symbolism have emerged from this hybrid method (see Table 10 for a summary). The CCA analysis revealed that the correspondences between sound and meaning can be identified in an intricately interwoven network of associations. It is particularly intriguing that, in both languages, some meaning features form semantic units larger than individual semantic features were mapped to clusters of sound properties. For example, “size” and “weight” were lumped together and associated with “voicing” in Japanese. In contrast, the cluster of “speed” and “energeticity” were together connected to “voicing” in English. These levels of abstraction for both meaning and sound features could not have been discovered by hypothesis-testing approaches like previous studies, which start with particular sound-symbolic associations to be tested.

**Table 10:** Summary of the findings.

Link	Phonetic feature	Mapping	Language
Link 1	C_1_ voiced	voiced = “heavy & big” / voiceless = “light & small”	J*+
Link 2	V_1_ height	low = “fast”	J*+
Link 3	C_1_ manner	nasal = “slow”	J*+
Link 4	C_1_ voiced	voiced = “slow & energy-less” / voiceless = “fast & energetic”	J+, E*
Link 5	C_1_ place	velar/palatal = “light & jerky”	J+, E*+

Note:

“*” indicates a link supported by CCA, and “+” indicates a link supported by the follow-up mixed-effects modelling. For languages, “J” stands for Japanese, and “E stands for English.

Importantly, the sound-symbolic links detected in the present study included both those that have already been reported in previous studies (e.g., voicing symbolism) and those that have not. This fact highlights the validity and usefulness of an exploratory approach to uncover the latent structure of sound-meaning correspondences.

Theoretically, the current results extended Dingemanse et al.’s proposal [7] that cross-linguistically shared and language-specific sound-meaning correspondences can co-exist within a single semantic domain. Interestingly, both English and Japanese adopted “voicing” as a primary phonetic feature for motion-sound symbolism. This sound-symbolic link might be attributed to the greater amplitude of voiced consonants compared to voiceless consonants, which appears to be easily mapped to the physical scales “speed” and “weight.” However, as noted earlier, how voicing was mapped to the meaning was somewhat varied across the two languages. Even when phonetic features used in sound symbolism are cross-linguistically shared, the way the sound is used to represent meanings depends on the language.

It is also worth noting that even if the same sound-symbolic links are found across languages, their relative significance might be different. We examined whether the sound-meaning links suggested by CCA were equally significant in the two languages. We found differences between English-based and Japanese-based sound symbolism in offering the cross-linguistically shared sound symbolism. The sound-meaning link identified in CCA in the English data was shared by Japanese speakers (Link 5, see Table 10), but the links found in the Japanese data (Links 1, 2, and 3) were not shared by the English speakers. It should be noted that one of the links (Link 4) found by CCA in the English group was found to be statistically reliable only in the Japanese group. This may look surprising but is possible considering that the sound-meaning associations were searched separately in the two languages,and that the strength of the connections between sounds and meanings was in general higher in the Japanese group than in the English group (see the canonical correlation values in the results of CCA). Thus, it is possible that the results from an exploratory approach and those from a hypothesis-testing approach do not completely agree, as they adopt different statistical algorithms. A hypothesis-testing approach is based on inferential statistics, and it examines whether independent variables significantly contribute to the dependent variables, but in CCA this is not the case. Therefore, the exploratory data-mining approach in general can present candidates for possible sound-meaning associations very broadly, as it detects all sound-meaning pairs which meet the pre-specified criteria. It is important to restrict the candidates in the exploratory data-mining step (e.g., by Tabachnick and Fidell’s criteria, as we did in the current study).

Why did we see language-specific sound-symbolic links only in the Japanese data? Here, it may be useful to draw on some semioticians’ proposal that iconicity can be divided into “primary” and “secondary iconicity” [20, 55]. Primary iconicity is readily perceivable without prior knowledge, whereas secondary iconicity requires the knowledge of conventional links between particular forms and meanings. The asymmetry found between the Japanese and English data may have arisen because English speakers exclusively relied on primary iconicity because English does not have elaborate and productive sound symbolic vocabulary as Japanese does. In contrast, it appears that the elaborate mimetic lexicon allowed Japanese speakers to detect both types of iconicity. In fact, a massive body of research has demonstrated that sound symbolism in Japanese constitutes a complex and highly conventionalized system with fine-grained semantic specifications. For example, mimetics for actions of cracking illustrate the common paradigms of vocalic and consonantal symbolism in the language: *p****o****kipoki* ‘cracking a one-dimensional object (e.g., a branch)’ vs. *p****a****kipaki* ‘cracking a two-dimensional object (e.g., a board)’; ***p****okipoki* ‘cracking a thin one-dimensional object’ vs. ***b****okiboki* ‘cracking a thick one-dimensional object [28]. These types of elaborate sound-meaning links embedded in a conventional lexical system of mimetics in a particular language may be examples of secondary iconicity, which would be difficult to sense or use without knowledge of the system.

In line with the present discussion, one may speculate that the “thinking for speaking” hypothesis partly explains how secondary iconicity influenced the labelling task in the current study [56]. Thinking for speaking refers to the idea that speakers pay special attention to features of the world that are needed or suitable for syntactic and lexical resources of the language as they verbalize their thoughts. For example, if your language has a tense system, you may pay attention to time in relation to the speech event. Similarly, it is possible that sound-symbolic links in the existing lexicon may shape the way we attend to various semantic features of an event. This may explain why English and Japanese showed slightly different structures of semantic dimensions in the present study.

### Iconicity and arbitrariness in language

The present results may also provide us with insights into a bigger issue: How important is iconicity in language? Traditionally, it has been assumed that linguistic symbols are amodal and arbitrary [1]. Sound symbolism provides evidence against this thesis. A rich body of literature has shown that sound-symbolic words are indeed more firmly grounded in sensory, perceptual or physical experiences [2, 57] than conventional, non-sound-symbolic words. Early researchers have assumed that such bodily basis is shared across languages, and hence, sound symbolism identified in one language should be available to speakers of other languages.

However, the results of the present study challenge this assumption, and suggest instead that sound symbolism is not limited to primary iconicity (see [20] for a similar view). The current study identified a few sound-symbolic links that are found in Japanese but not in English. This indicates that some sound-symbolic links in the Japanese data are based on secondary iconicity, which is likely to have emerged from the conventionalized, productive mappings between the form and meaning of Japanese mimetics. Such a possibility has long been underestimated because researchers have examined sound symbolism using a top-down hypothesis-testing approach. The present study has offered a new methodological paradigm that can expand the horizon of research on the nature of sound symbolism.

### Future work and methodological considerations

The method we proposed for the explorations of sound symbolism should be useful for semantic domains other than motion as well. It would be especially interesting to extend the present research to not-yet well studied domains such as texture, taste, color, emotion (cf. [7]), as well as to other languages to further investigate whether some semantic domains are more apt for sound symbolism than others across languages of the world. It would also be important to investigate whether there are sound-meaning mappings at an abstract level that go beyond individual semantic domains (e.g., abrupt change, regularity, stability, cf. [18, 24]).

Mainly due to the exploratory nature, the current study has some limitations. First, we forced English speakers to create sound-symbolic words in the CVCV template, which is not common in English. Although this decision was made for important reasons and that English-speakers’ recruitment of sound symbolism did not seem to have affected significantly (see page 11), we cannot rule out the possibility that this restriction affected the English speakers’ use of sound symbolism in some ways. It will be an interesting topic for future work to examine what kind of word template reduces the ease/difficulty of novel sound-symbolic word production. Second, though our study examined the sound-symbolic systems of Japanese and English as a test case for our proposed methodology, a fuller discussion on cross-linguistically shared and language-specific sound symbolism would need many more languages with various lexical background. Third, due to practical constraints in the analysis, we analyzed only word-initial segments, treating the C and V independently. Although it has been reported that the first syllable has the greatest significance in sound symbolism [38], it may also be possible that sound symbolism in the first and second syllables play different roles to create the sound-symbolic effects of the whole word (cf. [28]). Future research should explore whether different segments of a word jointly create sound-symbolic effects, and if so, how individual segments interacts with one another in sound symbolism. Fourth, we assumed that phonetic features are the explanatory primitives for sound symbolism. However, it is possible that a more abstract characterization of speech sound may provide more proper explanations. For example, in Japanese, nasal consonants and voiced consonants are both associated with slow movements. This may indicate that the concentration of acoustic energy in lower frequencies is associated with slowness.

## Supporting information

S1 File The actual instructions for the rating and production task in Japanese and English. (DOCX)
