# Supplementary material for: Cross-linguistically shared and language-specific sound symbolism in novel words elicited by locomotion videos in Japanese and English
Source: PLoS One. 2019 Jul 10;14(7):e0218707. doi: 10.1371/journal.pone.0218707 (PMC6619670; doi:10.1371/journal.pone.0218707)
Supplement: S1 File — (DOCX) [file pone.0218707.s001.docx]

The actual instructions for the attribute rating task in Japanese and English were as follows:

a. Japanese

*Ika no 70 no dooga o mite* (watch the following 70 videos), *sorezore no dooga no doosa ni taishite “ookisa,” “sokudo,” “karusa,” “kiryoku,” “namerakasa” no 5 no ten kara hyootei o okonatte kudasai* (rate the degree in which each of the following five features applies to each movement: “size,” “speed,” “weight,” “energeticity,” and “jerkiness”).

b. English

*Please watch the following 70 videos and rate the degree in which each of the following five features applies to each movement: “size,” “speed,” “weight,” “energeticity,” and “jerkiness.”*

The instructions for the production task were as follows:

a. Japanese

*Ika no 70 no dooga o mite* (watch the following 70 videos), *sorezore no dooga no doosa o arawasu to omou oto o motta atarashii go o hitotsuzutu tsukuridashite kudasai* (make up words whose sound you think matches the motion in the each video). *Go o tsukuru ni atatte wa* (in making up the words), *hutatsu no moora (shiin to boin no kumiawase) o mochiite kudasai* (use two moras consisting of a consonant and a vowel).

b. English

*Please watch the following movies and make up a new word whose sound intuitively matches the movement. The words should consist of two syllables where each syllable consists of a consonant followed by a vowel: for example, “baba,“ “toshi,” “nona,” “choro.” Please type in the word, then say the movie number (e.g., “Clip 23”) and pronounce the word loud.*
